# Effects of Core–Shell Heterogeneous Grain Structure Topology on Tensile Strength of CoCrFeMnNi High-Entropy Alloy Based on Crystal Plasticity Modeling

**DOI:** 10.3390/ma19122433

**Published:** 2026-06-07

**Authors:** Rubing Fu, Xin Wang, Zhe Zhang, Gang Chen

**Affiliations:** 1 School of Chemical Engineering and Technology, Tianjin University, Tianjin 300350, China; 2023207570@tju.edu.cn (R.F.); agang@tju.edu.cn (G.C.); 2 Department of Mechanical and Aerospace Engineering, Carleton University, Ottawa, ON K1S 5B6, Canada; xin.wang@carleton.ca

**Keywords:** core–shell structure, CoCrFeMnNi high-entropy alloy, crystal plasticity finite element method, hetero-deformation induced hardening, strength

## Abstract

Heterogeneous grain structured design has emerged as an effective strategy to overcome the limitations of mechanical properties in structural materials. Core–shell heterogeneous grain structured materials exhibit a good strength-ductility synergy owing to their continuously networked grain topology. However, controlling the grain size and fraction in core–shell structures through mechanical milling and powder metallurgy remains challenging. Therefore, the effects of grain structure topology on mechanical behavior remain unclear. This study establishes a crystal plastic finite element (CPFE) model of a core–shell structure and discusses the effects of core–shell topological characteristics, i.e., core–shell fraction (S_f_ = 15% to 65%), the core–shell interface gradient (θ = 63° to 90°), and the coarse grain/ultrafine grain size ratio (CG/UFG = 8/2 to 8/1), on tensile strength and hetero-deformation induced (HDI) hardening. The results indicate that the tensile strength is strongly correlated with the core–shell fraction and CG/UFG size ratio. The tensile strength is enhanced with increasing core–shell fraction and CG/UFG size ratio, which can be attributed to the increased fraction of ultrafine grains and their reduced grain size. The tensile strength increases by approximately 30% when the core–shell fraction increases from 15% to 65%, and increases by approximately 12% when the CG/UFG size ratio changes from 8/2 to 8/1. However, these two parameters exhibit a negligible influence on HDI hardening. In contrast, compared to θ = 63°, the HDI hardening at θ = 90° increases by approximately 20%, thus it indicates the sharp core–shell interface gradient markedly promotes HDI hardening, thereby improving the tensile strength through an increased hardening rate. Collectively, this study provides useful information for the microstructure design of core–shell heterogeneous grain structured materials.

## 1. Introduction

Enhancing tensile strength is an important pathway to achieving structural safety, reliability, extended service life, lightweight design, and reduced carbon emissions [1,2,3]. Currently, various typical heterostructures have been developed, including gradient structure (GS) [4,5], multimodal structure (MS) [6,7,8,9], lamellar structure (LS) [10,11], and core–shell heterogeneous grain structure (i.e., harmonic structure, HS) [12,13,14]. Among these, the core–shell structure consists of a coarse-grained core region and a three-dimensional ultrafine/nanocrystalline network shell region [15,16,17]. Typically, it is prepared by subjecting initial metal powders to severe plastic deformation (SPD), such as mechanical ball milling and high-energy jet milling, followed by sintering via hot isostatic pressing (HIP) or spark plasma sintering (SPS). Extensive research demonstrates that the core–shell structure exhibits superior tensile strength, fatigue limit, and impact resistance compared to homogeneous grain structured materials, highlighting the critical role of grain structure topology in deformation behavior. In addition to improving tensile strength, the network ultrafine grain structure also effectively suppresses fatigue crack initiation and enhances fatigue life [18].

Experimental studies have provided important insights into the deformation mechanism of the core–shell structures. For example, micro-digital image correlation (Micro-DIC) analyses by Park [19] and Li et al. [20] revealed significant strain heterogeneity between the core and shell regions. In addition, in situ X-ray diffraction studies by Sjögren-Levin et al. [21] revealed distinct elastic–plastic transition behavior and stress evolution in heterogeneous regions. The core–shell structure exhibits pronounced stress and strain inhomogeneity, which originates from the strain gradients generated between the soft and hard regions. Notably, geometrically necessary dislocations (GNDs) accumulate near heterogeneous interfaces at densities much higher than those in homogeneous grain structured materials, thereby playing a dominant role in heterostructure strengthening [22,23,24]. Zhu et al. [25,26,27] proposed the hetero-deformation induced (HDI) hardening and strengthening mechanisms for this phenomenon. Most current studies suggest that the HDI stress plays a crucial role in enhancing the mechanical properties of heterogeneous grain structured materials [28,29,30].

In recent years, with the rapid development of crystal plasticity finite element (CPFE) methods, researchers have gradually gained deeper insight into the deformation mechanisms of heterogeneous grain structured materials [31,32,33]. Notably, these studies have further enhanced the capability of the simulation to capture complex deformation behaviors by incorporating mechanisms related to grain size effects, grain boundary resistance, and HDI hardening [34,35,36,37,38,39]. For the core–shell structures, numerical simulation has become an important tool for revealing deformation mechanisms and guiding microstructural design. Early studies predominantly employed multiscale homogenization or mean-field models to reduce the computational cost associated with directly modeling three-dimensional core–shell structures. For instance, Liu et al. [40] developed a three-dimensional multiscale model based on crystal plasticity and homogenization, incorporating the Hall-Petch grain size effect and damage evolution. Wang et al. [41,42] further applied this framework to predict the monotonic and cyclic shear behavior of harmonic-structured Ti. However, although such models mainly focus on the effective representation of macroscopic stress–strain behavior, the effects of microstructure features on local strain accumulation, HDI hardening, and macroscopic mechanical properties remain insufficiently understood. Park et al. [43] considered core grain size, shell grain size, and core–shell fraction as key structural variables, and combined an HDI strengthening model with machine learning to optimize core–shell structures. While this approach aims to capture the complex microstructure–property relationships in core–shell systems, the strong coupling of multiple structural variables in the model makes it difficult to distinguish the independent contributions of different topological parameters to yield strengthening, strain hardening, and HDI hardening. Zhang et al. [34,44] investigated the effects of shell fraction and heterogeneous interface density on the mechanical behavior of CoCrFeMnNi high-entropy alloys using an improved crystal plasticity model. They found that increasing shell fraction and interface density significantly enhances yield strength, ultimate tensile strength, and back stress, while the strain hardening rate shows an approximately positive correlation with interface density. Yuan et al. [45] further coupled this model with a cohesive zone model, revealing the role of back stress in promoting crack-tip blunting, as well as the contribution of an optimal fine-grained shell ratio to enhancing fracture resistance. However, it is insufficient to fully capture the influence of core–shell interface gradient and the size mismatch between coarse grain (CG) and ultrafine grain (UFG) on local strain gradient and HDI hardening behavior.

Although most existing works have primarily focused on individual structural parameters [46,47,48], such as grain size, core–shell fraction, or strength mismatch, the quantitative roles of topology features remain insufficiently explored. In particular, the continuous gradient across heterogeneous core–shell interface has not yet been adequately considered in reported studies. Furthermore, owing to the limitations of fabrication processes, the evolution of core–shell microstructure involves complex coupling effects, making it difficult to independently control grain size, shell fraction, and interface gradient characteristics. Consequently, experimental approaches face significant challenges in quantitatively evaluating the influence of specific topological parameters on the mechanical properties of materials. In contrast, CPFE simulations can effectively decouple structural variables, thereby providing an effective approach for investigating the effects of different topological characteristics on strength and HDI hardening behavior. Therefore, this work establishes a CPFE model for core–shell structured CoCrFeMnNi high-entropy alloys (HEAs), calibrated by experimental results and high-resolution digital image correlation (HR-DIC) strain field measurements. The effects of core–shell fraction, core–shell interface gradient, and coarse grain/ultrafine grain size ratio on strain distribution, HDI hardening behavior, and tensile strength are systematically investigated. This study is expected to provide theoretical support for a deeper understanding of the deformation mechanisms in core–shell heterogeneous grain structured materials.

## 2. Materials and Methods

### 2.1. Generation of Core–Shell Microstructures

**Figure 1:**
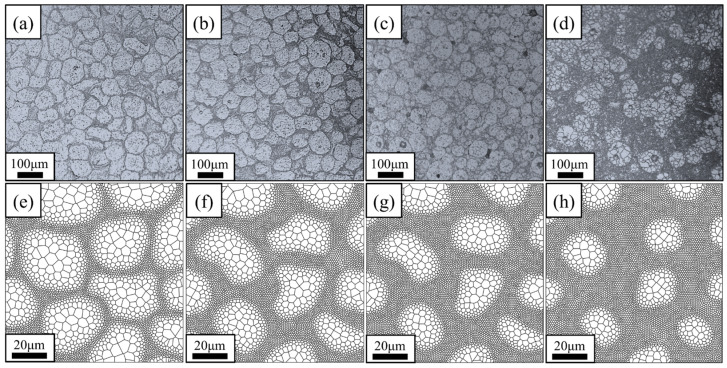
Optical microstructures and model of core–shell structure with varying grain size (**d**) and core–shell fraction S_f_: (**a**,**e**) d_core_ = 12.5 μm, d_shell_ = 1.4 μm, S_f_ = 15%; (**b**,**f**) d_core_ = 11.0 μm, d_shell_ = 1.4 μm, S_f_ = 45%; (**c**,**g**) d_core_ = 10.1 μm, d_shell_ = 0.8 μm, S_f_ = 50%; (**d**,**h**) d_core_ = 9.5 μm, d_shell_ = 1.1 μm, S_f_ = 65%.

Core–shell structured CoCrFeMnNi HEA compacts are fabricated by mechanical milling followed by SPS, as shown in Figure 1. The optical micrographs in Figure 1a–d reveal a pronounced bimodal grain size distribution, in which CGs (core region) are embedded in UFG network structure (shell region). The core–shell fractions (S_f_) of the four samples are approximately 15%, 45%, 50% and 65%, respectively. The computational models are constructed using a Voronoi algorithm combined with triangular mesh seed points through the following two steps:

(1) The core–shell regions and interfaces are determined, and different numbers of mesh seeds are placed in different regions;
(2) Triangular meshes are generated, and the vertices of the interconnected meshes serve as the initial positions for Voronoi tessellation to generate grains.

**Figure 2:**
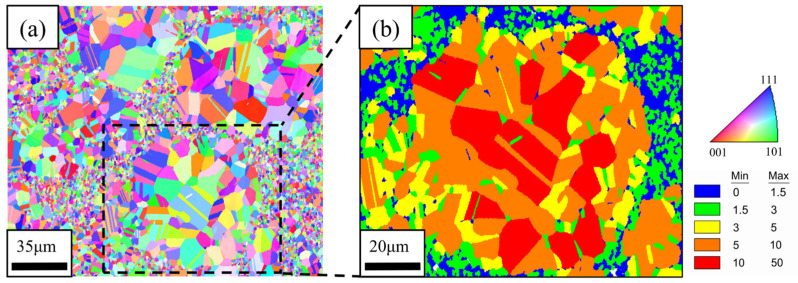
(**a**) IPF of the core–shell structure; (**b**) magnified view of the grain size distribution.

Through Voronoi tessellation, a polycrystalline morphology with a core–shell structure was constructed, exhibiting a transition in grain size, as shown in Figure 1e–h. This method can construct various desired core–shell structure by controlling the size of the core–shell region and the number of meshes. Figure 2a presents the inverse pole figure (IPF) and grain size distribution of the typical sample. The material consists of equiaxed grains with a random orientation distribution. Some annealing twins can also be observed in the CG or UFG regions. The distribution of annealing twins is irregular, and their amount is relatively small compared with that of ultrafine grains. The weak boundary-strengthening contribution that they may provide has been equivalently incorporated into the model through experimentally calibrated parameters. Therefore, consistent with similar studies, the effects of annealing twins and twin boundaries are not explicitly considered in the reported works [44,45]. Figure 2b further illustrates the local microstructural features. The shell region consists of equiaxed UFGs with grain sizes smaller than 3 μm, whereas the core region consists of equiaxed CGs with grain size larger than 3 μm. A continuous grain size gradient is observed between shell and core regions.

### 2.2. Crystal Plasticity Constitutive Model

#### 2.2.1. Kinematics

In this study, a constitutive model describing core–shell structured materials is developed using the CPFE. This model is combined with experimental results to quantitatively analyze the mechanical response of RVEs based on the material’s microstructure [49,50]. Based on a phenomenological crystal plasticity slip-rate model, a hardening model based on dislocation-density evolution and a kinematic hardening model describing HDI hardening are implemented in Abaqus (ver 2022) as a user material subroutine (UMAT) [51,52,53].

According to crystal kinematics theory, deformation encompasses modes associated with lattice elastic stretching, lattice rotation and plastic slip [54,55]. The total deformation gradient tensor F can be decomposed as the product of elastic $F_{e}$ and plastic $F_{p}$ deformation gradient tensor components:

(1) $$F=F_{e}F_{p},$$

where $F_{e}$ involves the stretching and rotation of the atomic lattice; while $F_{p}$ relates solely to the slip of dislocations within the crystal and represents the plastic deformation gradient of the intermediate configuration. Assuming that elastic properties remain unaffected by slip, and that the lattice orientation remains identical to that of the reference configuration, the rate of change of $F_{p}$ is related to the slip rate ${\dot{\gamma }}^{a}$ of the slip system *a*.

(2) $${\dot{F}}_{p}\cdot F_{p}^{-1}=\sum_{a}{\dot{\gamma }}^{a}s_{0}^{a}⊗n_{0}^{a},$$

In the deformed configuration, the slip direction $s^{a}$ and slip plane normal vector $n^{a}$ for slip system *a* are as follows:

(3) $$s^{a}=F_{e}s_{0}^{a},n^{a}=F_{e}^{-T}n_{0}^{a},$$

In the deformed configuration, the velocity gradient L can be expressed via the deformation gradient tensor as:

(4) $$L=\dot{F}\cdot F^{-1}=D+\Omega =\frac{1}{2}\left(L+L^{T}\right),$$

where the symmetric rate of stretching **D** and the antisymmetric spin tensor **Ω** may be decomposed into elastic parts and plastic parts as follows:

(5) $$D=D_{e}+D_{p},\Omega =\Omega_{e}+\Omega_{p},$$

The sum of the rates of change in the elastic and plastic velocities is expressed in terms of the respective rates of change in the deformation gradient. Specifically, the rate of change in the deformation gradient is related to the slip rate of slip system *a* in the deformed configuration, as well as the slip direction vector $s^{a}$ and the slip plane normal vector $n^{a}$, and is expressed as:

(6) $$D_{e}+\Omega_{e}={\dot{F}}_{e}\cdot F_{e}^{-1},D_{p}+\Omega_{p}=\sum_{a}{\dot{\gamma }}^{a}s_{0}^{a}⊗n_{0}^{a},$$

The elastic stretching rate tensor $D_{e}$ and the elastic rotation rate tensor $\Omega_{e}$ are expressed in terms of the deformation gradient as:

(7) $$D_{e}=\frac{1}{2}\left({\dot{F}}_{e}F_{e}^{-1}+\left(F_{e}^{-1}\right){\dot{F}}_{e}^{T}\right),\Omega_{e}=\frac{1}{2}\left({\dot{F}}_{e}F_{e}^{-1}-\left(F_{e}^{-1}\right){\dot{F}}_{e}^{T}\right),$$

#### 2.2.2. Constitutive Laws

According to the objective stress rate constitutive equation proposed by Hill and Rice [56]:

(8) $$\sigma_{e}^{\nabla }+\sigma \mathrm{tr}\left(D_{e}\right)=\mathbb{C}:D_{e},$$

where $\sigma_{e}^{\nabla }$ denotes the Jaumann objective stress rate of the Cauchy stress with respect to the crystal coordinate system; σ is the Cauchy stress; and ℂ is the fourth-order elastic tensor, for cubic materials possessing three independent elastic constants—C_11_, C_12_, and C_44_—it is directly represented in the form of a 6 × 6 matrix:

(9) $$\left[\mathbb{C}\right]=\left[\begin{array}{cccccc} C_{11} & C_{12} & C_{12} & 0 & 0 & 0\\ C_{12} & C_{11} & C_{12} & 0 & 0 & 0\\ C_{12} & C_{12} & C_{11} & 0 & 0 & 0\\ 0 & 0 & 0 & C_{44} & 0 & 0\\ 0 & 0 & 0 & 0 & C_{44} & 0\\ 0 & 0 & 0 & 0 & 0 & C_{44} \end{array}\right],$$

The relationship between $\sigma_{e}^{\nabla }$ and the Cauchy stress $\dot{\sigma }$ rate is given by:

(10) $$\sigma_{e}^{\nabla }=\dot{\sigma }-\Omega_{e}\sigma +\sigma \Omega_{e},$$

The relationship between Jaumann objective stress rate of Cauchy stress with respect to the material coordinate system $\sigma^{\nabla }$ and $\dot{\sigma }$:

(11) $$\sigma^{\nabla }=\dot{\sigma }-\Omega \sigma +\sigma \Omega ,$$

Therefore, combining Equations (5), (10) and (11), the relationship between $\sigma^{\nabla }$ and $\sigma_{e}^{\nabla }$ is:

(12) $$\sigma^{\nabla }=\sigma_{e}^{\nabla }-\Omega_{p}\sigma +\sigma \Omega_{p},$$

Furthermore, the plastic stretching rate tensor $D_{p}$ and the plastic rotation rate tensor $\Omega_{p}$ can also be obtained through symmetric decomposition using Equation (4).

#### 2.2.3. Slip and Hardening Laws

Slip rate ${\dot{\gamma }}^{a}$ is calculated using the commonly employed power-law slip model, employing the resolved shear stress as the driving force for slip and the critical resolved shear stress (CRSS) as the threshold for slip initiation, as shown in the following formula:

(13) $${\dot{\gamma }}^{a}={\dot{\gamma }}_{0}^{a}{\left(\frac{\left|\tau^{a}-\chi^{a}\right|}{\tau_{\mathrm{ceff}}^{a}}\right)}^{n}\mathrm{sgn}\left(\tau^{a}-\chi^{a}\right),$$

where ${\dot{\gamma }}_{0}^{a}$ and n denote the reference slip rate and rate sensitivity index, respectively. $\chi^{a}$ represents the kinematic hardening term, formulated according to the Armstrong-Frederick kinematic hardening model [57]:

(14) $${\dot{\chi }}^{a}{=h}_{b}{\dot{\gamma }}^{a}-\frac{r_{D}}{\tau_{\mathrm{ceff}}}\chi^{a}\left|{\dot{\gamma }}^{a}\right|,$$

The above equation represents the kinematic hardening equation under the crystal plasticity framework, where $h_{b}$ and $r_{D}$ denote the hardening term and dynamic recovery term parameters of the model, respectively. According to the paper published by Zhang [34] and Yuan [45], the differences in strength and size between coarse and fine grains induce a plastic deformation gradient in the vicinity of the heterogeneous interface, thereby promoting the accumulation of GNDs to accommodate this gradient; the GNDs density $\rho_{\mathrm{GNDs}}$ is directly proportional to the ratio of the plastic deformation gradient $\eta^{p}$ to the Burgers vector b:

(15) $$\rho_{\mathrm{GNDs}}\propto \frac{\eta^{p}}{b},$$

Considering the Hall-Petch stress relationship, it can be approximately expressed in terms of the stress mismatch between adjacent grains:

(16) $$\eta^{p}\approx ∆\sigma =\sigma_{d_{n}}-\sigma_{d}{=k}_{b}\left(\frac{1}{\sqrt{d_{n}}}-\frac{1}{\sqrt{d}}\right),$$

where *d* is the current grain size, $d_{n}$ is the average grain size of the surrounding grains, and $k_{b}$ is the Hall-Petch slope. Drawing upon strain gradient theory, the flow stress is correlated with the density of GNDs through the Taylor hardening relationship:

(17) $$\sigma =\mu b\sqrt{\rho_{\mathrm{GNDs}}}=\mu \sqrt{{\mathrm{bk}}_{b}\left(\frac{1}{\sqrt{d_{n}}}-\frac{1}{\sqrt{d}}\right)}\approx k_{b}\left(\frac{1}{\sqrt{d_{n}}}-\frac{1}{\sqrt{d}}\right),$$

Heterogeneous deformation significantly influences flow stress and manifests a more pronounced Bauschinger effect; therefore, it is appropriate to incorporate the HDI stress component into the kinematic hardening model. The kinematic hardening term is thus reformulated as a function of the grain size and the sizes of its adjacent neighbors, aiming to quantify the extent of local HDI hardening:

(18) $$h_{b}=h_{b}^{*}+k_{b}\langle \frac{1}{\sqrt{d_{n}}}-\frac{1}{\sqrt{d}}\rangle ,$$

Equations (13) and (14) define $\tau_{\mathrm{ceff}}^{a}$ as the effective critical resolved shear stress, which incorporates the effects of the Hall-Petch effect and dislocation density evolution:

(19) $$\tau_{\mathrm{ceff}}^{a}=\left(\frac{\tau_{0}+\frac{k_{s}}{\sqrt{d}}}{M}\right){+\mathrm{Gb}}^{a}\alpha \sqrt{\rho^{a}},$$

the first part denotes $\tau_{0}$, $k_{s}$, and d as the initial CRSS, Hall-Petch slope, and grain size, respectively. The second part denotes G, α, and $b^{a}$ as the shear modulus, geometric factor, and Burgers vector of slip system *a*, respectively. The evolution of dislocation density follows the Kocks-Mecking-Estrin (KME) model [58], which accounts for mutual obstruction between dislocations arising from nucleation and annihilation. The hardening term is inversely proportional to the square root of dislocation density, while the softening term is directly proportional to dislocation density:

(20) $${\dot{\rho }}^{a}=\left(k_{1}\sqrt{\rho^{a}}-k_{2}\rho^{a}\right)\left|{\dot{\gamma }}^{a}\right|,$$

$k_{1}$ is the dislocation multiplication coefficient, and $k_{2}$ is the dislocation annihilation coefficient. The HEA studied in this work is the FCC crystal structure, with {111}⟨110⟩ slip systems as the primary deformation mode.

### 2.3. Determination of Model Parameters

**Figure 3:**
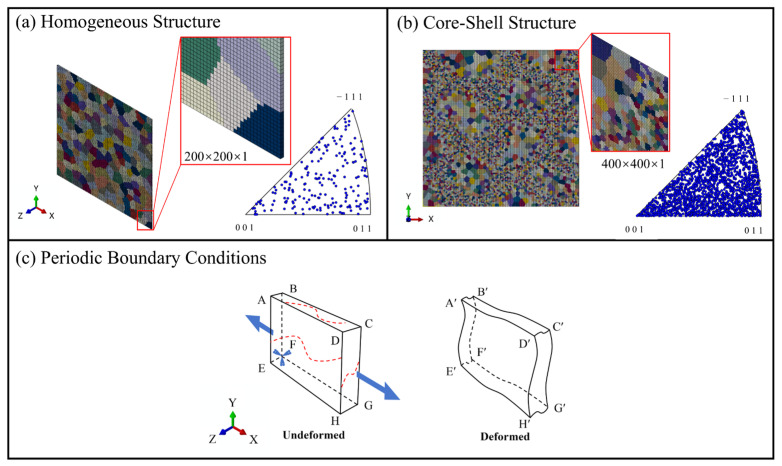
(**a**) Polycrystalline model of homogeneous structure (Texture index: 1.183); (**b**) core–shell structure with mesh partitioning (Texture index: 1.018); (**c**) periodic boundary conditions applied to quasi-3D model and schematic illustration of boundary changes after deformation.

**Figure A1:**
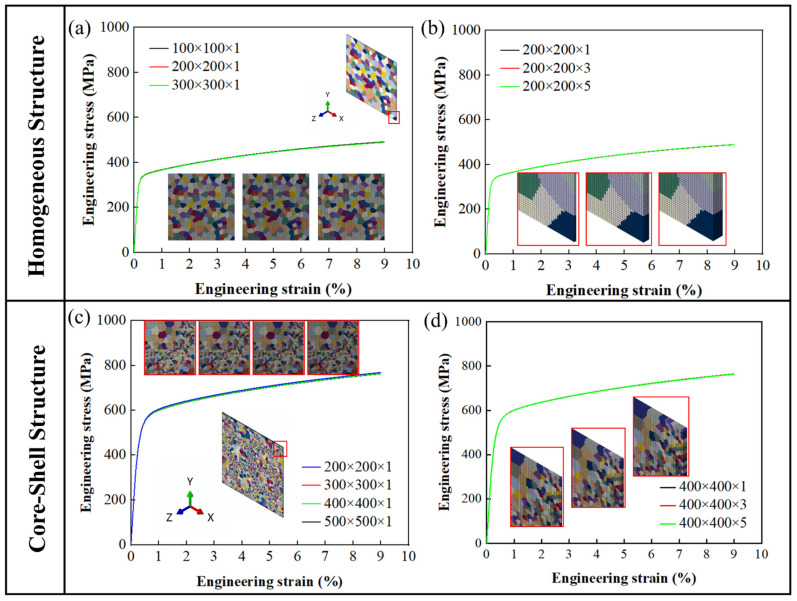
Sensitivity analysis of (**a**) mesh count and (**b**) model thickness for the homogeneous structure; sensitivity analysis of (**c**) mesh count and (**d**) model thickness for the core–shell structure (The area within the red box is the region selected in the model for magnification.).

Considering finite element computational efficiency, the mesh density is subject to certain limitations. To ensure parameter consistency between homogeneous grain structure and core–shell structure, all models in this study adopt a quasi-3D modeling approach, with grains mapped using C3D8 elements. Such models have been shown to avoid the overestimation of local strain inherent in 2D models and to serve as efficient substitutes for 3D models [59]. However, its degrees of freedom in the thickness direction remain constrained, making it difficult to fully characterize the true 3D grain morphology and the complex stress–strain coordination behavior [34,59,60,61]. The mesh density and the sensitivity analysis of the number of mesh layers in the thickness direction are presented in Figure A1 in Appendix A. The selected mesh configurations are relatively conservative, and the stress–strain response curves under the reference condition exhibit good consistency. Therefore, the final mesh numbers adopted in this study, 200 × 200 × 1 for the homogeneous grain structure and 400 × 400 × 1 for the core–shell structure, represent a balanced compromise between computational accuracy and computational cost. The finite element model employed is illustrated in Figure 3a,b. With the crystal orientation Euler angles set to random values, the IPF shown in Figure 3a,b demonstrate that both the uniform and core–shell structures exhibit random crystallographic orientation distribution in their crystal orientations, with texture indices of 1.183 and 1.018, respectively (where a value closer to 1 indicates stronger randomness).

The application of boundary conditions is illustrated using the ABFE and DCGH surfaces of the model shown in Figure 3c. The periodic boundary conditions applied are expressed as follows [62]:

(21) $$U_{Y}^{\mathrm{ABFE}}-U_{Y}^{\mathrm{DCGH}}=0,$$

(22) $$U_{Z}^{\mathrm{ABFE}}-U_{Z}^{\mathrm{DCGH}}=0,$$

(23) $$U_{X}^{\mathrm{ABFE}}-U_{X}^{\mathrm{DCGH}}-U_{X}^{\mathrm{RP}}=0,$$

where RP denotes a reference point established within the space. As shown in Equation (23), the displacement of this reference point in the x-direction represents the relative displacement between the ABFE and DCGH faces along the x-direction. Therefore, to achieve uniaxial tensile deformation of the model along the x-direction, an x-direction displacement load can be applied at the reference point. Similar periodic boundary conditions are applied to the other faces. Furthermore, adding periodic boundaries alone cannot guarantee that the model will not undergo rigid body displacement during deformation. Therefore, displacement constraints in three directions must also be applied at point F. The model before and after deformation under this boundary condition is shown in Figure 3c.

**Figure 4:**
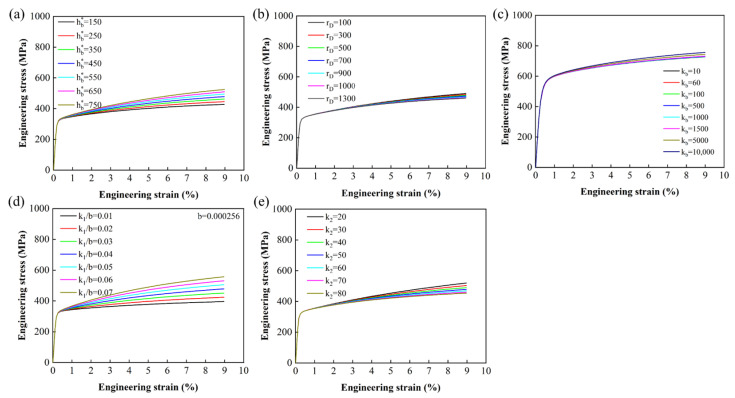
(**a**–**c**) Stress–strain response for different values of HDI hardening model parameters $h_{b}$, $r_{D}$, $k_{b}$; (**d**,**e**) dislocation density hardening model parameters $k_{1}$, $k_{2}$.

The strain rate constant ${\dot{\gamma }}_{0}^{a}$, n, elastic constants $C_{11}$, $C_{12}$, $C_{44}$, Burgers vector $b^{a}$, and polycrystalline cluster Taylor factor M can all be obtained from previous studies [54,63,64,65]. The reference value ranges for the dislocation multiplication parameter $k_{1}$ and dislocation annihilation parameter $k_{2}$ in the dislocation strengthening model are obtained from Refs. [66,67]. The Hall-Petch slope $k_{s}$ and initial lattice resistance $\tau_{0}$ are obtained from references [68,69] as initial values; the hardening term constant $h_{b}^{*}$, dynamic recovery constant $r_{D}$, and HDI hardening parameter of the kinematic hardening model are obtained from references [70,71] as reference values. Figure 4 presents the variations in the stress–strain response under different parameter values. Appropriate parameters sets are determined through a trial-and-error calibration procedure based on these variation trends. The sensitivity analysis indicates that the HDI hardening parameters and dislocation multiplication parameters strongly influence strain hardening behavior and local strain partitioning, whereas the elastic parameters and some recovery-related parameters have comparatively limited influence on the overall simulation results. Therefore, the present study mainly focused on the calibration and optimization of the highly sensitive parameters. Grain size is characterized via Electron backscatter diffraction (EBSD) experiments, with an overall range of 0.1–50 μm. Simultaneously, grain number, grain orientation, and grain size are input as the material’s information. Neighboring grain number and size for each grain are obtained during the preprocessing stage by traversing elements via a Python (ver 3.11.9) script.

**Figure 5:**
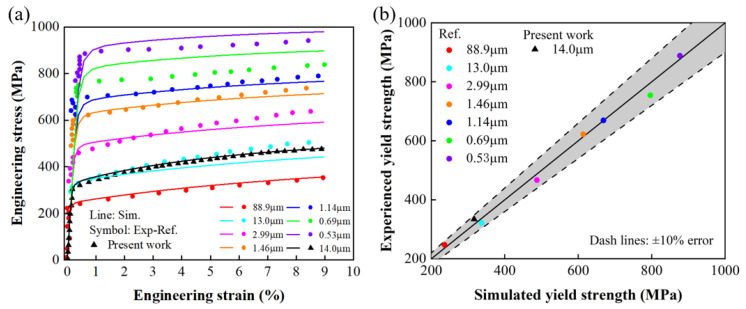
(**a**) Fitting of the Hall-Petch effect for yield strength under 9% strain loading at different grain sizes; (**b**) Error in simulated yield strength [71,72].

The yield strength of homogeneous grain structured CoCrFeMnNi HEAs with various grain sizes is calibrated using a homogeneous model. The fitting results for parameters related to the elastic stage and yield strength are shown in Figure 5a. The calibrated Hall-Petch slope and intercept enable uniaxial tensile data at various grain sizes to agree with experimental results. Figure 5b demonstrates that all yield strength errors are within a 10% tolerance band. Specifically, the black triangular data points represent the experimental results obtained in this study, while the remaining data are sourced from the literature. The subtle discrepancies in strain-hardening characteristics between the two datasets are attributed to minor variations in the material preparation process; consequently, the strain-hardening data derived from the experiments conducted in this study are utilized for all subsequent simulation calculations.

**Figure 6:**
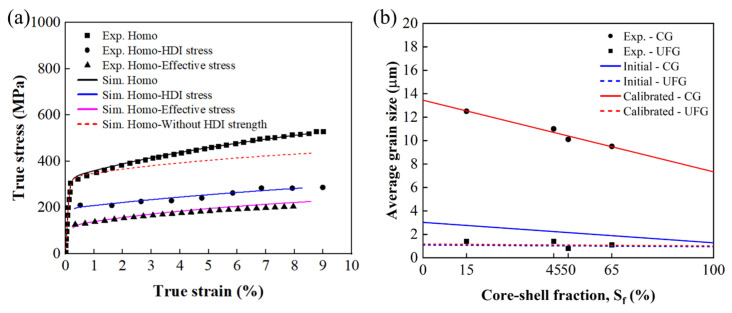
(**a**) Representative fitting of the effective stress $\sigma_{\mathrm{eff}}$ and HDI stress $\sigma_{\mathrm{HDI}}$ for the homogeneous grain structure; (**b**) comparisons of the initial model average grain size, experimental average grain size, and calibrated average grain size.

Hardening parameters for the homogeneous grain structured material are determined based on an average grain size of 14 μm. The calibration process is illustrated in Figure 6a. Specifically, the dislocation hardening parameters, kinematic hardening parameters, and HDI hardening parameters are gradually adjusted so that the simulated results are consistent with the experimental results in terms of yield strength, flow stress level, strain hardening behavior, and HDI stress evolution trend. Parameter calibration is considered to reach convergence when the overall error between the simulated and experimental curves is controlled within approximately 1–5%, and further parameter adjustment produced only minor changes in the simulation results. From uniaxial tensile and LUR test results, the effective stress curve $\sigma_{\mathrm{eff}}$ and HDI stress curve $\sigma_{\mathrm{HDI}}$ are obtained via the following equations [72]:

(24) $$\sigma_{\mathrm{HDI}}=\frac{\sigma_{u}+\sigma_{r}}{2},$$

(25) $$\sigma_{\mathrm{eff}}=\sigma_{f}-\sigma_{\mathrm{HDI}},$$

where $\sigma_{f}$ represents the overall flow stress under unloading strain, while $\sigma_{u}$ and $\sigma_{r}$ denote the yield stresses during the LUR process, respectively. The effective stress reflects the degree of dislocation strengthening, primarily originating from the isotropic resistance caused by lattice friction and local pinning at the grain boundary [73]. To fit the core–shell structure parameters and systematically investigate the influence of S_f_ on mechanical behavior, four representative core–shell structures are constructed based on experimental results. Due to limitations in computational scale and mesh density, the grain size distribution generated by geometry deviates from the experimental results. The UFGs in the shell are consistent with the experimental results, but the average grain size in the core layer is smaller. To ensure that the simulated microstructure scale accurately reflects the experimental material, the initial grain size of the core is increased and redistributed to match the experimental distribution. Figure 6b shows the average grain size of the experimental results and the initial average grain size of the model, as well as the final average grain size adopted by the model after calibration. The calibrated grain statistics are consistent with the grain size distribution observed in the experiment. This strategy provides a reliable microstructure basis for subsequent mechanical response analysis.

**Figure 7:**
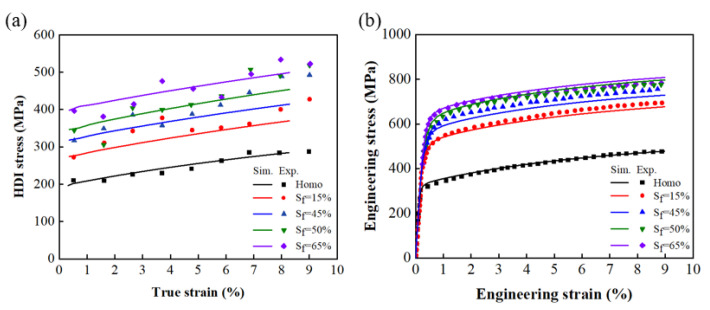
(**a**) Fitted HDI stress-true strain curves for different shell fractions; (**b**) Fitted engineering stress–strain responses for homogeneous and core–shell structures.

By simultaneously fitting both curves, the dislocation hardening parameters and HDI stress parameters for the homogeneous grain structure are determined, as shown in Figure 7a,b. To ensure the accuracy of deformation simulation in the CPFE model, the local deformation field of the core–shell structure obtained using HR-DIC technology is employed for comparison. The specific steps are as follows:

(1) A nanoscale speckle pattern is prepared on the specimen surface to enable strain tracking. This is achieved through ion sputter deposition followed by chemical etching (Speckles are fabricated using Ag target material via ion sputter deposition at 30 mA for 60 s, followed by etching in 1% NaCl solution for 10 min to produce nanoscale silver speckles 50–100 nm).
(2) Near in situ scanning electron microscopy (SEM) images are acquired before and after applying 1% tensile strain. The corresponding strain fields are then calculated using the open-source software Ncorr (ver 1.3).
(3) Based on EBSD characterization of the same region, the CPFE model is constructed by assigning grain orientations and sizes, enabling a direct comparison between the simulated and experimental strain fields.

**Figure 8:**
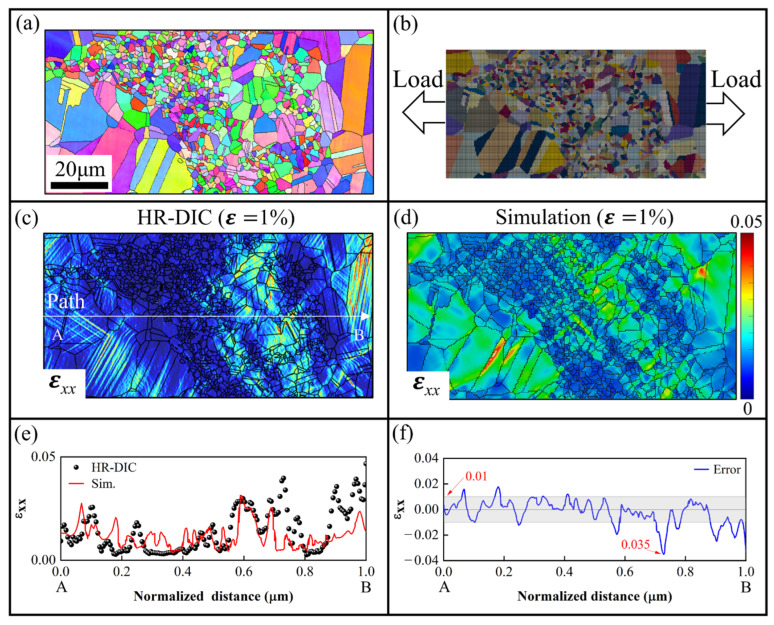
(**a**,**b**) Modeling of the core–shell structure based on EBSD results; (**c**,**d**) comparison of simulation results with HR-DIC strain fields at 1% tensile strain; (**e**,**f**) comparison of strain values along the path (in (**c**)), and the error curve (The line from A to B in the figure represents the selected path, and the gray band indicates the strain range of ±0.01).

Figure 8a,b shows the EBSD microstructure and the mesh model of the modeling region, respectively. Considering the computational cost, a relatively coarse mesh is adopted. However, its influence on the strain distribution is limited. As shown in Figure 8c,d, the simulated strain field agrees well with the HR-DIC measurements at a strain level of 1%. Along the same path shown in Figure 8c, approximately 80% of the data points fall within a strain error margin of 0.01. Only a few points show errors larger than 0.01, with a maximum strain error of 0.035. Nevertheless, these discrepancies may still influence the prediction accuracy of local strain concentration. Notably, the primary objective of the HR-DIC comparison at 1% strain is to validate the ability of the present CPFE model to reproduce local deformation. Then, the hardening parameters for the core–shell structure are obtained from the results of uniaxial tension and LUR tests. Figure 8d shows the final simulation results demonstrated good agreement with the experimental data. The corrected material parameters are listed in Table 1.

**Figure 9:**
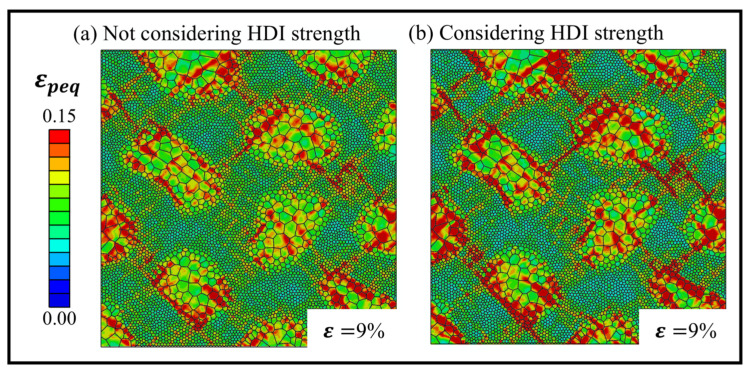
Simulation results of equivalent plastic strain fields in core–shell structure with S_f_ = 50% for 9% tensile strain: (**a**) not considering HDI strength; (**b**) considering HDI strength.

To evaluate the role of HDI stress in the mechanical behavior of materials, simulations are conducted considering or not considering the contribution of HDI stress by enabling and disabling the kinematic hardening term in Equation (14). The equivalent plastic strain fields of the S_f_ = 50% structure at 9% strain are shown in Figure 9. Compared with the model not considering HDI strength, the model incorporating HDI exhibits a more uniform strain distribution. High-strain localization becomes weaker and more spatially dispersed, rather than remaining concentrated mainly in the core region. Instead, more coordinated deformation occurs between the core and shell regions. These results demonstrate the constraining effect of HDI stress on local plastic deformation. HDI stress suppresses excessive plastic flow in soft zones and promotes more coordinated deformation between the core and shell regions [34]. This finding further confirms the important contribution of HDI hardening to the enhanced plastic deformation capacity of core–shell structure at the microscale. Furthermore, the mutual interactions among the different microstructural parameters are analyzed (see Appendix B). The hardening rate curves indicate that each parameter exerts a relatively independent influence on the deformation behavior.

## 3. Results and Discussion

### 3.1. Effect of Core–Shell Fraction on Tensile Strength

**Figure 10:**
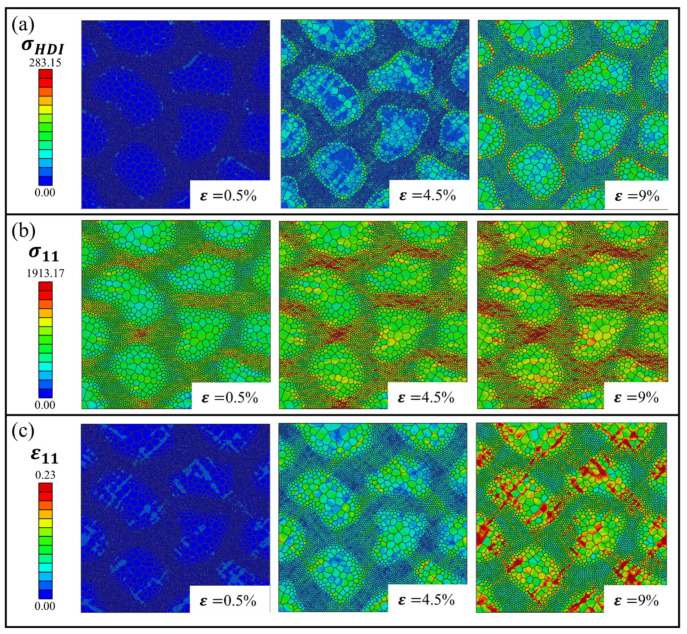
Typical HDI strength in the models with S_f_ = 45%: (**a**) HDI stress field; (**b**) stress field of x direction; (**c**) strain field of x direction.

This section investigates the influence of the S_f_ on the strengthening behavior. S_f_ is varied from 15% to 65%, while maintaining a constant core–shell interface gradient and CG/UFG size ratio consistent with experimental conditions. Figure 10a shows the HDI stress distribution for a typical core–shell structure with S_f_ = 45%. At the initial stage of deformation, (ε = 0.5%) HDI stress first develops near the core–shell interface. With increasing deformation, it progressively intensifies at the heterogeneous interface and increases markedly within the shear bands in both core and shell regions. The simulation results are consistent with previous reports on HDI hardening in heterogeneous grain structured materials [33,60,68]. Moreover, HDI stress is closely associated with the GNDs accumulation at the interface. With continuously increasing strain, the inhomogeneous distribution of dislocations further amplifies the local HDI stress. Figure 10b,c shows the evolution of the stress and strain fields along the loading direction (x-direction) at different strain levels (0.5%, 4.5% and 9%). Noticeable inhomogeneous stress distribution is observed. High-stress zones form in the narrow shell region, while the interior of the core exhibits relatively dispersed stress. A low-stress channel appears vertically to the loading direction between shell region and core region [61]. This reflects stress concentration caused by load transfer and geometric constraints. Strain localization not only tends to develop at a 45°to the tensile direction but is also dispersed near the core–shell interface.

**Figure 11:**
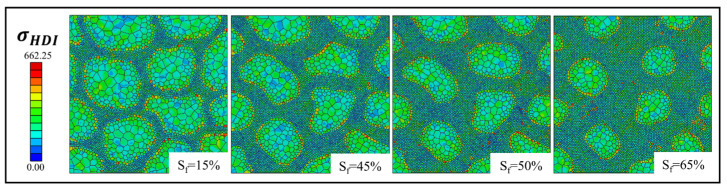
Simulation results for HDI stress field of the models with different S_f_ at ε = 9%.

Figure 11 compares the HDI stress in different core–shell structures at ε = 9%. Consistent with the above results, pronounced HDI stress accumulation is observed near the core–shell interface, whereas only minor differences appear in the local stress distribution among different S_f_ values. This suggests that, under the present grain size gradient conditions, HDI hardening is relatively insensitive to S_f_. When the grain sizes and interface gradient remain nearly unchanged, the strain mismatch across the interface is also largely preserved. As a result, the GNDs density required to accommodate the strain gradient does not vary substantially, leading to a similar level of HDI stress development. Consequently, the evolution of HDI strengthening remains nearly unchanged even with increasing S_f_. This phenomenon is consistent with the macroscopic HDI hardening behavior observed in the stress–strain responses (see Figure 7a).

**Figure 12:**
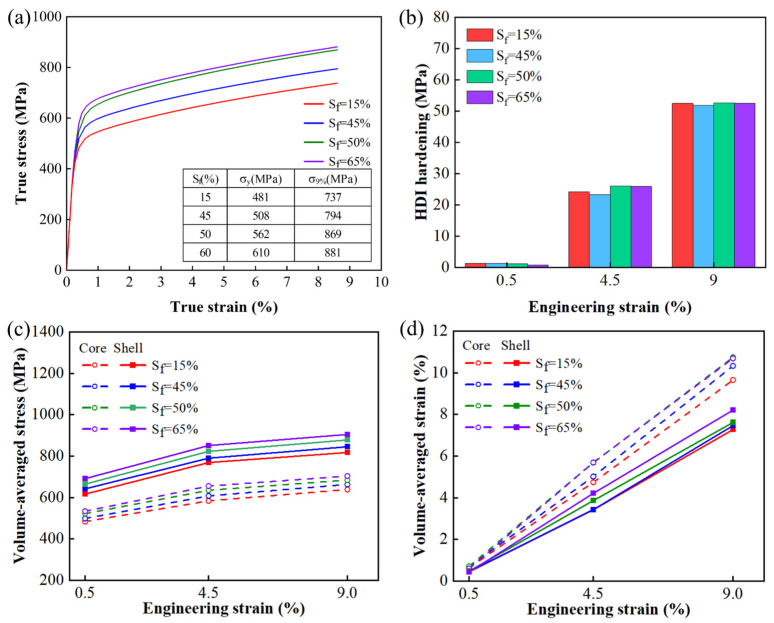
Tensile behavior in the models with different S_f_: (**a**) stress–strain response curve; (**b**) HDI hardening; (**c**) volume-averaged stress; (**d**) volume-averaged strain.

Figure 12 illustrates the effect of S_f_ on tensile strength and HDI hardening behavior. The uniaxial tension simulation results in Figure 12a show that the yield strength increases with increasing S_f_. As the S_f_ increases from 15% to 65%, the strength increases by approximately 30%. This trend originates from the increased fraction of ultrafine grains (UFGs) in the shell region, where finer grains provide stronger resistance to dislocation motion and thus enhance the strength. In contrast, S_f_ has a limited effect on strain hardening. As shown in Figure 12b, the HDI hardening term defined in Equation (14) is used to characterize the accumulation of HDI, because it is proportional to strain and can clearly reflect the evolution of HDI with increasing strain. the strain-hardening rates of the models are nearly identical at the same strain. This behavior can be attributed to the fact that strain hardening in heterogeneous grain structured materials is dominated by HDI hardening, which is primarily controlled by strain gradients rather than the S_f_ alone. Under the present conditions, the strain incompatibility between the core and shell regions changes only slightly with increasing S_f_; therefore, the resulting GNDs accumulation and HDI stress evolution remain similar.

The HDI hardening increases with tensile strain in all models, reflecting the progressive accumulation of GNDs and the continuous development of HDI stress during deformation, but its variation among different S_f_ values remains limited. Figure 12c,d present the volume-averaged strain and stress distribution at strains of 0.5%, 4.5%, and 9%. The average stress in the shell region is significantly higher than that in the core region, whereas the corresponding strain is lower. This stress–strain partitioning arises from the strength contrast between the harder shell and the relatively softer core. As tensile strain increases, the deformation mismatch promotes progressive strain localization in the core region. In addition, the strain accumulation rate in the core region increases significantly with increasing core–shell fraction, indicating enhanced deformation partitioning between the core and shell regions.

### 3.2. Effect of Core–Shell Interface Gradient on Tensile Strength

**Figure 13:**
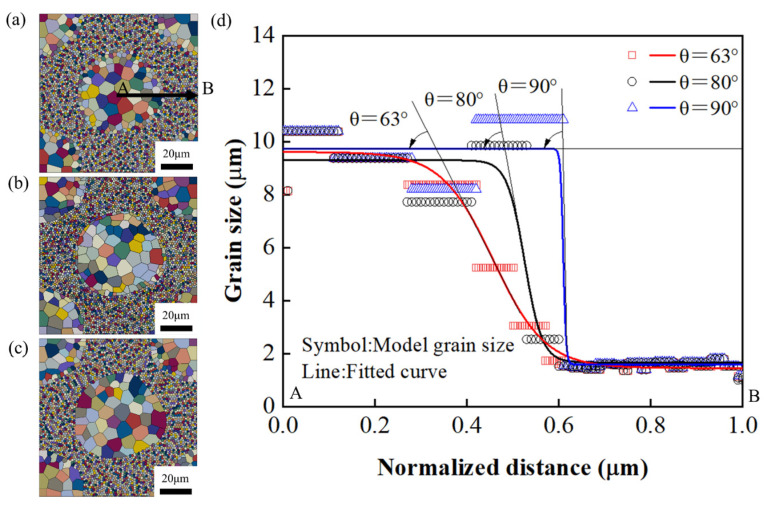
Modeling of different core–shell interface gradients: (**a**) θ = 63°; (**b**) θ = 80°; (**c**) θ = 90°; (**d**) Core–shell interface gradient along the AB path.

This section explores the effects of interface gradient on strengthening behavior of the core–shell structured materials. The models with different interface gradients are established at a fixed core–shell fraction of 45%, as shown in Figure 13. The UFG and CG sizes are maintained at approximately 1.5 μm and 10 μm, respectively. Due to the presence of the interface gradient, slight variations in UFG and CG sizes among the three models are unavoidable. However, their influence on strain hardening is negligible. Figure 13a–c compare three core–shell structure models with different interface gradients, while Figure 13d presents the grain size distribution along the AB path. The angle between the interface gradient direction and horizontal direction is defined as θ, where a larger θ corresponds to a sharper core–shell interface. Based on the EBSD experimental results presented in Appendix C, the model with θ = 63° represents the experimental condition. In this work, θ is varied from 63° to 90°.

**Figure 14:**
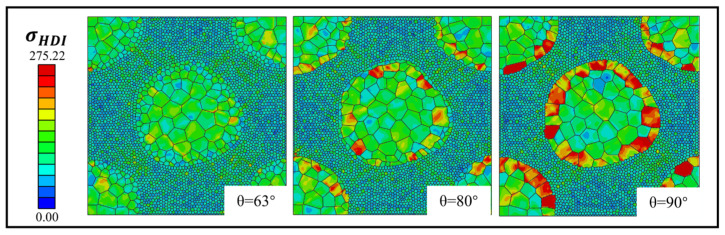
Simulation results for HDI stress field of the models with different core–shell interface gradients at ε = 9%.

Figure 14 illustrates the HDI stress fields in the models with different interface gradients under the same elongation. As θ increases, the core–shell interface becomes sharper, leading to a stronger HDI stress concentration at the interface. This behavior arises from the more abrupt spatial variation in mechanical properties across the interface, which enhances local deformation incompatibility and promotes HDI stress accumulation. In contrast, the interface in the θ = 63° model alleviates strain localization and reduces HDI hardening. Like gradient structured materials, the interface gradient enables smoother deformation transfer between regions with different strengths, thereby reducing severe strain discontinuities, delaying localized deformation, and promoting more uniform plastic deformation.

**Figure 15:**
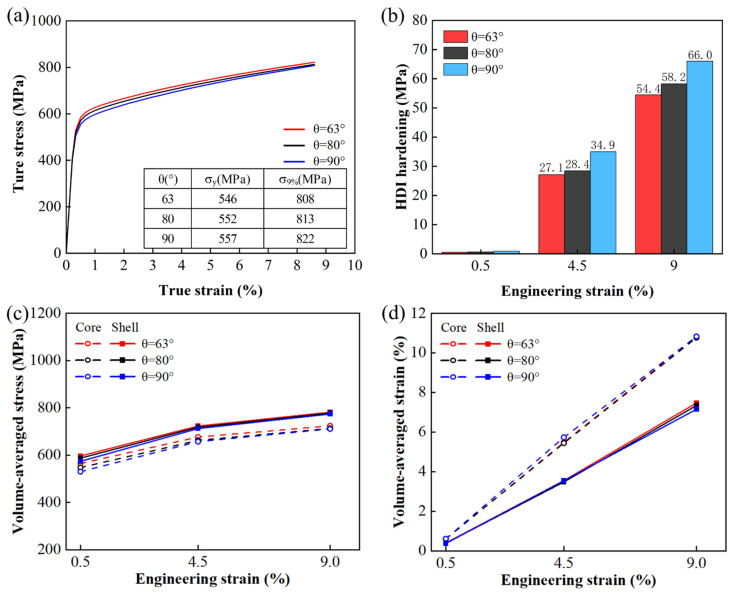
Tensile behavior in the models with different core–shell interface gradients: (**a**) stress–strain response curve; (**b**) HDI hardening; (**c**) volume-averaged stress; (**d**) volume-averaged strain.

Figure 15a shows the simulated true stress-true strain responses of models with different core–shell interface gradients. The θ = 63° model exhibits a slightly higher yield strength, which can be attributed to the relatively smaller average grain size in its core region. Since yield strength is mainly governed by the overall resistance to dislocation motion, it is strongly affected by the average grain size. All three models show continuously increasing strain hardening during tensile deformation, reflecting the progressive evolution of internal stress fields. As shown in Figure 15b, HDI hardening increases with increasing θ. Compared with θ = 63° model, the HDI hardening increment at θ = 90° is approximately 20% higher. This trend originates from the enhanced strain incompatibility at the sharp interface, which intensifies the storage of GNDs and the development of long-range HDI stress. Figure 15c,d further illustrate the volume-averaged strain and stress distributions at strains of 0.5%, 4.5%, and 9%. Although the average strain and stress increase with tensile strain, the differences between the core and shell regions change only slightly with increasing θ. This suggests that the global stress–strain partitioning is relatively insensitive to the interface gradient. Instead, the θ mainly affects local deformation near the interface and the macroscopic strain hardening response. As θ increases, the interface becomes sharper, causing strain gradients to localize within a narrower region.

### 3.3. Effect of CG/UFG Size Ratio on Tensile Strength

**Figure 16:**
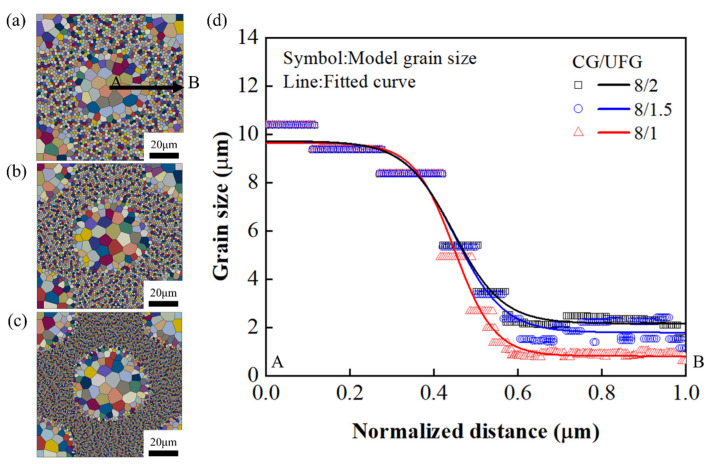
Models with different CG/UFG size ratios: (**a**) CG/UFG = 8/2; (**b**) CG/UFG = 8/1.5; (**c**) CG/UFG = 8/1; (**d**) grain size distribution along the AB path.

This section investigates the effect of CG/UFG size ratio (μm/μm) on tensile behavior. Under the condition that the core–shell fraction (S_f_ = 45%) and the interface gradient are kept constant and consistent with experimental observations, the CG/UFG size ratio is varied from 8/2 to 8/1. As shown in Figure 16a–c, the models are constructed with identical core and shell regions, while only the UFG size is varied to achieve the grain size ratios of CG/UFG = 8/2, 8/1.5 and 8/1, respectively. The corresponding grain size transition profiles along the AB path are presented in Figure 16d.

**Figure 17:**
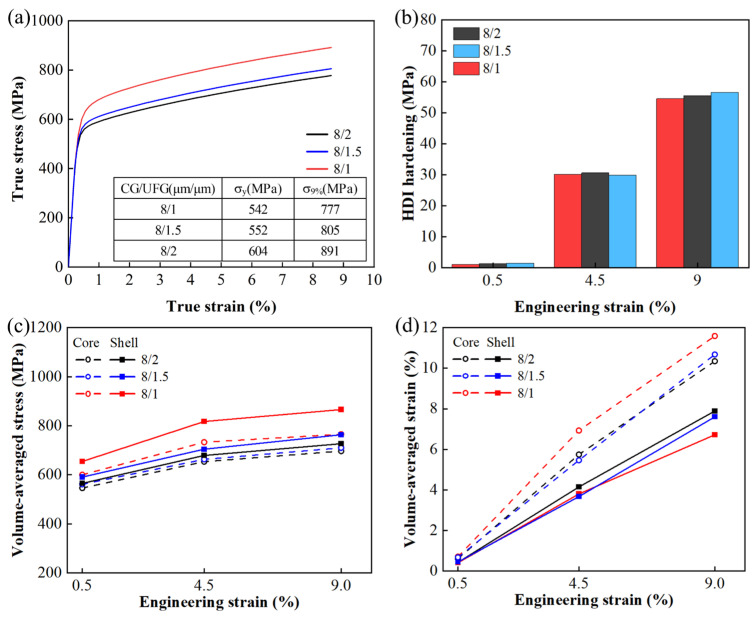
Tensile behavior in the models with different CG/UFG size ratios: (**a**) Stress–strain response curve; (**b**) HDI hardening; (**c**) volume-averaged stress; (**d**) volume−averaged strain.

**Figure A5:**
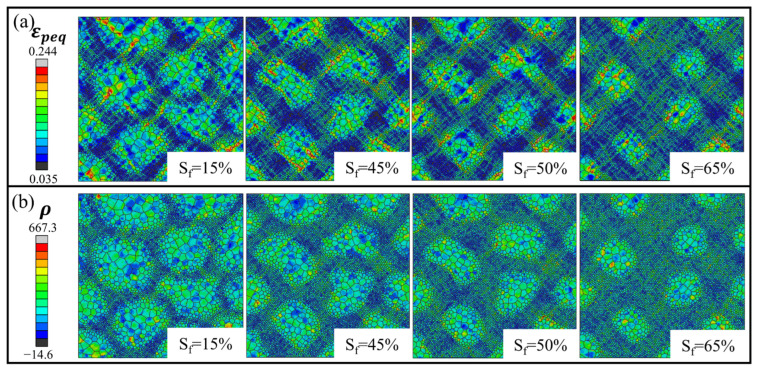
Deformation distribution in the models with different S_f_: (**a**) Equivalent plastic strain field; (**b**) dislocation density field.

**Figure A6:**
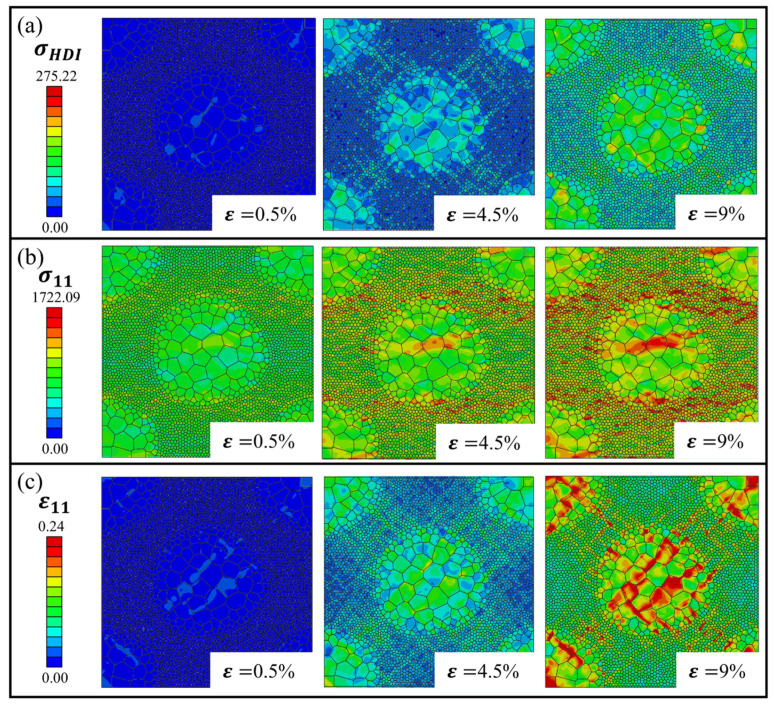
Typical HDI strength in the models with the interface gradient θ = 63° at different strains: (**a**) HDI stress field; (**b**) stress field of x direction; (**c**) strain field of x direction.

**Figure A7:**
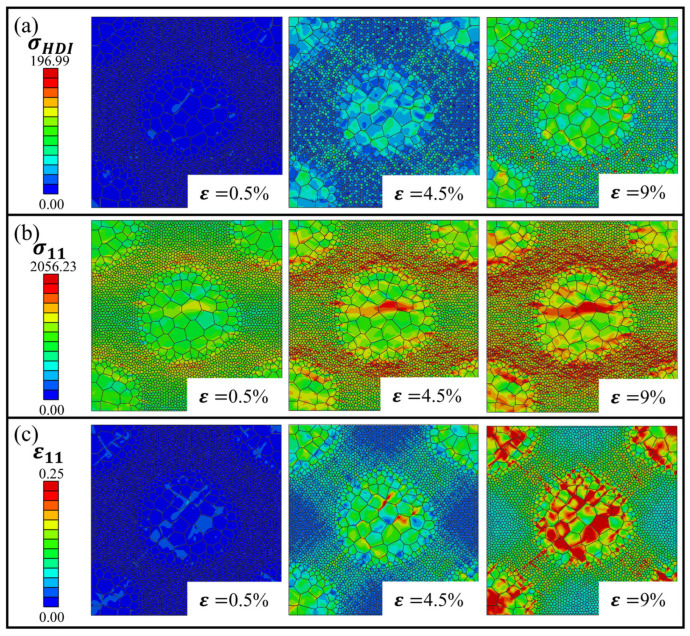
Typical evolution of HDI stress in the models with CG/UFG = 8/1: (**a**) HDI stress field; (**b**) stress field of x direction; (**c**) strain field of x direction.

**Figure A8:**
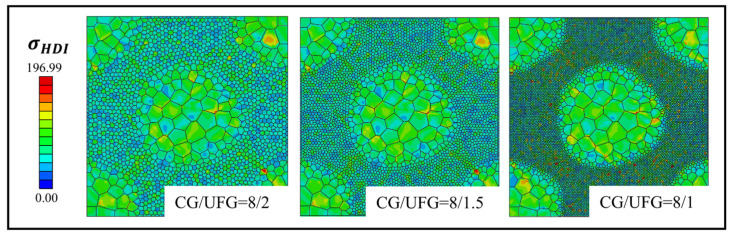
Simulation results of HDI stress fields in the models with different CG/UFG size ratios at ε = 9%.

Figure 17a presents the simulated stress–strain responses of the three models. The yield strength increases as the CG/UFG size ratio changes from 8/2 to 8/1, with an increase of approximately 12%. This trend is consistent with enhanced grain-boundary strengthening caused by the reduced average grain size. However, all three models exhibit nearly identical strain hardening rates, indicating that variations in the CG/UFG size ratio have only a minor influence on strain hardening behavior. Figure 17c,d show the volume-averaged stress and strain distribution at strains of 0.5%, 4.5%, and 9%. Although both values increase with increasing tensile strain, their increments differ significantly between the two regions. With increasing CG/UFG size ratio, the increase in volume-averaged stress in the shell becomes more pronounced, whereas the increase in volume-averaged strain is greater in the core. This asymmetric evolution reflects the redistribution of deformation between the two regions, promoting stress concentration in the harder shell and strain accommodation in the softer core. However, this redistribution mainly affects the deformation partitioning rather than the magnitude of strain gradients at the interfaces. Consequently, although the CG/UFG size ratio strongly influences the spatial pattern of deformation accumulation, its effect on HDI strengthening remains limited, because the fundamental mechanisms governing GNDs generation and HDI stress development are not substantially altered. Other related field variables are presented in Figure A5, Figure A6, Figure A7 and Figure A8 in Appendix D.

Although the present CPFE model can effectively describe heterogeneous deformation and HDI hardening behavior in core–shell heterogeneous grain structures, it is still based on a quasi-3D modeling approach. Therefore, some deviations from real three-dimensional microstructures and grain topologies remain, limiting its capability to characterize complex three-dimensional stress states and spatial deformation coordination behavior [60]. In addition, the present study mainly proposes design strategies for improving tensile strength, whereas the microstructural parameters may also significantly affect crack initiation and propagation. To achieve a better strength-ductility synergy in core–shell structured materials, further work should incorporate damage-evolution models, such as CPFE coupled with cohesive elements [75], continuum damage mechanics models [76], or phase-field fracture constitutive models [77], to more comprehensively reveal the strength-ductility synergy mechanisms and service behavior of heterogeneous HEAs.

## 4. Conclusions

This study investigated the tensile behavior of heterogeneous grain structured CoCrFeMnNi HEA using the crystal plasticity finite element (CPFE) method. The model parameters are derived from tensile tests and HR-DIC strain measurement. The effects of core–shell structure topological characteristics (i.e., core–shell fraction, core–shell interface gradient and core–shell grain size ratio) on tensile strength, strain hardening behavior and hetero-deformation induced (HDI) hardening behavior are analyzed. The main conclusions are summarized as follows:

(1) The strength of the core–shell structure is collectively influenced by the core–shell fraction, interface gradient, and CG/UFG size ratio. As the core–shell fraction increases, the proportion of the UFG increases, leading to a significant enhancement in the overall tensile strength. In addition, increasing the CG/UFG size ratio also contributes to enhance strength. However, these two parameters exhibit only a limited influence on strain hardening. In contrast, a sharper interface gradient further enhances the HDI hardening and strain-hardening capability of the material, thereby improving the overall strength.
(2) The three topological parameters also show significant influences on the heterogeneous deformation distribution of the material. As the core–shell fraction increases, the UFG shell region bears higher stress, while the stronger constraint on the core region leads to a faster increase in strain, resulting in a more non-uniform strain distribution between the two regions. As the interface gradient becomes sharper, the local deformation incompatibility near the core–shell interface becomes more pronounced. Increasing CG/UFG size ratio further intensifies the mismatch between the core and shell regions, thereby affecting the macroscopic spatial distributions of stress and strain.
(3) Strength can be effectively enhanced by increasing the core–shell fraction, sharpening the interface gradient, and simultaneously maintaining a relatively large CG/UFG size ratio, thereby reasonably promoting the HDI hardening mechanism. However, the non-uniform local stress–strain distribution may also affect crack initiation and propagation behavior. In particular, the heterogeneous interface is more likely to become a potential damage-concentration region. Therefore, the effects of different topological parameters on plastic deformation and fracture behavior need to be further investigated for good strength-ductility synergy.

## Figures and Tables

**Table 1:** Crystal plasticity material parameters.

Parameter	Definition	Value	Refs.
${\dot{\gamma }}_{0}^{a}$	Reference shear rate	0.001	[72,73]
n	Rate sensitivity constant	20	[72,73]
$b^{a}$	Burgers vector in slip system a $\left(\mu m\right)$	0.000256	[55,74]
$C_{11}$	Normal elastic stiffness constant $\left(\mathrm{MPa}\right)$	264,000	[73]
$C_{12}$	Elastic coupling constant $\left(\mathrm{MPa}\right)$	184,000	[73]
$C_{44}$	Shear elastic constant $\left(\mathrm{MPa}\right)$	112,000	[73]
$k_{1}$	Dislocation density multiplication constant $\left({\mu m}^{-1}\right)$	156.25	fitted
$k_{2}$	Dislocation annihilation constant	40	fitted
α	Taylor strength geometric factor	0.2	fitted
$k_{s}$	Hall-Petch slope $\left(\mathrm{MPa}\cdot {\mu m}^{-1/2}\right)$	590	fitted
$\tau_{0}$	Initial lattice friction resistance $\left(\mathrm{MPa}\right)$	291	fitted
M	Taylor factor for a polycrystal aggregate	3.1	[72]
$h_{b}^{*}$	Kinematic hardening constant MPa	450	fitted
$r_{D}$	Dynamic recovery constant MPa	500	fitted
$k_{b}$	HDI hardening constant $\left(\mathrm{MPa}\cdot {\mu m}^{-1/2}\right)$	5000	fitted

**Figure A2:**
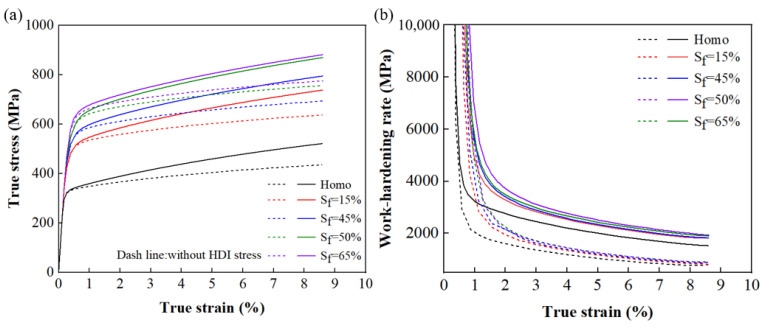
(**a**) True stress-true strain curves and (**b**) strain hardening rate curves for the models with different S_f_, considering and not considering HDI strength. It can be observed that, when considering HDI hardening, the overall distribution of the strain rate curves aligns with the trends observed when HDI hardening is not taken into account. Specifically, the variations in grain size distribution resulting from different values of S_f_ did not significantly alter the strain hardening level predicted by the model; this indicates that the strain hardening behavior across these various models is primarily governed by the interface gradient.

**Figure A3:**
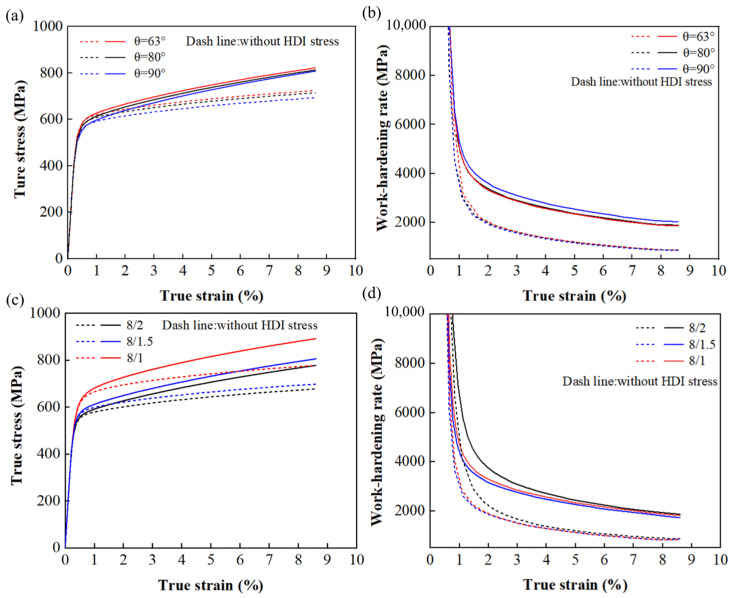
(**a**,**b**) True stress-true strain curves and strain hardening rate curves for the models with different θ, considering and not considering HDI strength. (**c**,**d**) True stress-true strain curves and strain hardening rate curves for the models with different CG/UFG size ratios, considering and not considering HDI strength. A comparative analysis reveals that, in the model with θ = 63°, the overall distribution of the strain-hardening rate curve—when HDI hardening is taken into account—remains consistent with the trend observed when HDI hardening is disregarded. The strain-hardening rate curve exhibits a significant rise only when the interface gradient is particularly abrupt and the value of θ is large; this indicates that the strain hardening in these various models is primarily governed by the gradient at the heterogeneous interface.

**Figure A4:**
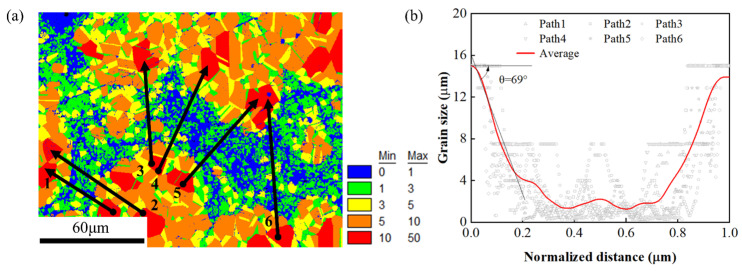
(**a**) EBSD grain size distribution map of the core–shell structure and selected paths; (**b**) grain size distribution and average grain size distribution for each path.

## Data Availability

The original contributions presented in this study are included in the article. Further inquiries can be directed to the corresponding author.

## Abbreviations

The following abbreviations are used in this manuscript:

GS	Gradient structure
MS	Multimodal structure
LS	Lamellar structure
HS	Harmonic structure
SPD	Severe plastic deformation
HIP	Hot isostatic pressing
SPS	Spark plasma sintering
Micro-DIC	Micro-digital image correlation
GNDs	Geometrically necessary dislocations
HDI	Hetero-deformation induced
CPFE	Crystal plasticity finite element
CG	Coarse grain
UFG	Ultrafine grain
HEAs	High-entropy alloys
HR-DIC	High-resolution digital image correlation
IPF	Inverse pole figure
RVEs	Representative volume elements
UMAT	User material subroutine
CRSS	Critical resolved shear stress
KME	Kocks-Mecking-Estrin
FCC	Face-centered cubic
LUR	Load-unload-reload
EBSD	Electron backscatter diffraction
SEM	Scanning electron microscopy

## Appendix A. Mesh and Parameter Sensitivity

Appendix A presents variations in the dislocation density hardening parameters and HDI hardening parameters within specific value ranges in Figure A1.

## Appendix B. The Independence of Heterogeneous Interface Gradient and Grain Size Effects

This section presents supplementary data for the models calculated in Section 3, “Results and Discussion”. Its purpose is to demonstrate that the strain hardening influenced by the discussed HDI hardening, and the yield strength influenced by grain size, constitute independent mechanisms with minimal mutual interaction. The data includes true stress-true strain curves and strain hardening rate curves for models considering and not considering the HDI strength.

## Appendix C. Grain Size Distribution in Interface Gradient

Appendix C provides the EBSD basis for modeling the core–shell interface gradient described in Section 3.2. To accurately characterize the gradient in the experimental core–shell structured materials, multiple representative paths are selected along the transition direction from the core to the shell region in the EBSD grain size distribution map. These paths are used to analyze the continuous variation in grain size across the shell layer between two coarse-grained core regions, as shown in Figure A4. After averaging the grain size distributions along six paths, a clear linear decrease is observed in the transition region from coarse grains to ultrafine grains. The angle between the interface gradient direction and the horizontal direction is determined to be θ = 63°. This parameter experimentally reflects the intensity of the abrupt grain size change at the core–shell interface, providing a basis for establishing a heterogeneous structure model consistent with the experimental results.
